# β-sitosterol induces G1 arrest and causes depolarization of mitochondrial membrane potential in breast carcinoma MDA-MB-231 cells

**DOI:** 10.1186/1472-6882-13-280

**Published:** 2013-10-25

**Authors:** Shanthi Sri Vundru, Raosaheb K Kale, Rana P Singh

**Affiliations:** 1 School of Life Sciences, Central University of Gujarat, Gandhinagar, Gujarat, India; 2 Cancer and Radiation Biology Laboratory, School of Life Sciences, Jawaharlal Nehru University, New Delhi, India

**Keywords:** Lung cancer, Skin cancer, Breast cancer, Cell proliferation, Cell death, Cell cycle and apoptosis

## Abstract

**Backgrounds:**

It is suggested that dietary phytosterols, such as β-sitosterol (ST), have cancer chemopreventive effects; however, studies are limited to support such claims. Here, we evaluated the efficacy of ST on three different human cancer cell lines including skin epidermoid carcinoma A431 cells, lung epithelial carcinoma A549 cells and breast adenocarcinoma MDA-MB-231.

**Methods:**

Cell growth assay, cell cycle analysis, FACS, JC-1 staining, annexin V staining and immunoblotting were used to study the efficacy of ST on cancer cells.

**Results:**

ST (30–90 μM) treatments for 48 h and 72 h did not show any significant effect on cell growth and death in A431 cells. Whereas similar ST treatments moderately inhibited the growth of A549 cells by up to 13% (p ≤ 0.05) in 48 h and 14% (p ≤ 0.05-0.0001) in 72 h. In MDA-MB-231 cells, ST caused a significant dose-dependent cell growth inhibition by 31- 63% (p ≤ 0.0001) in 48 h and 40-50% (p ≤ 0.0001) in 72 h. While exploring the molecular changes associated with strong ST efficacy in breast cancer cells, we observed that ST induced cell cycle arrest as well as cell death. ST caused G0/G1 cell cycle arrest which was accompanied by a decrease in CDK4 and cyclin D1, and an increase in p21/Cip1and p27/Kip1 protein levels. Further, cell death effect of ST was associated with induction of apoptosis. ST also caused the depolarization of mitochondrial membrane potential and increased Bax/Bcl-2 protein ratio.

**Conclusions:**

These results suggest prominent *in vitro* anti-proliferative and pro-apoptotic effects of ST in MDA-MB-231 cells. This study provides valuable insight into the chemopreventive efficacy and associated molecular alterations of ST in breast cancer cells whereas it had only moderate efficacy on lung cancer cells and did not show any considerable effect on skin cancer cells. These findings would form the basis for further studies to understand the mechanisms and assess the potential utility of ST as a cancer chemopreventive agent against breast cancer.

## Background

Among many cancers, breast cancer in females and lung cancer in males are the most frequently diagnosed cancers and the leading cause of cancer death for each sex in both economically developed and developing countries [1]. Lung cancer accounted for 13% of the total cases and 18% of the deaths due to cancer occurred in 2008 [1]. Skin cancer is the major cutaneous malignancy and about 76,250 people are estimated to be diagnosed for skin cancer in 2013 [2]. Incidence rate for melanoma has been rising from past three decades. Breast cancer is by far the most frequent cancer among women with an estimated 23% of all cancers and is the most frequent cause of cancer death in women [1].

The use of natural, synthetic or biological agents to prevent, reverse or suppress the growth and progression of cancer is referred as chemoprevention of cancer [3]. It is one of the most promising strategies for cancer control, and is accomplished by various means including chemoprevention by phytochemicals from vegetables, fruits, spices, teas, herbs and medicinal plants thus making it as one of the most feasible means of cancer control [4]. Phytochemicals are secondary plant metabolites and have been used for centuries throughout the world in traditional cures and herbal remedies, and as ayurvedic and homeopathic medicines in India. More recently, there has been a considerable interest in secondary plant metabolites because of their potential preventative effects on chronic diseases including cancer.

Many studies have demonstrated that phytochemicals in common fruits and vegetables can have complementary and overlapping mechanisms of action, including antioxidant activity, scavenging free radicals and regulation of gene expression, including oncogenes and tumor suppressor genes, in cell proliferation and cell differentiation; induction of cell-cycle arrest and apoptosis; modulation of enzyme activities in detoxification, oxidation and reduction; stimulation of the immune system; regulation of hormone metabolism; and antibacterial and antiviral effects [5-7]. Phytosterols (PS) are triterpenes that are important structural components of plant membranes. More than 200 different types of phytosterols have been reported in plant species. The richest sources of phytosterols are vegetable oils, nuts, cereal products, fruits and berries [8]. Structural resemblance of PS with cholesterol enables them to displace low-density lipoprotein (LDL) cholesterol in the human intestine [9]. Protective effects of PS against cardiovascular diseases (CVD), colon and breast cancer developments have been widely documented. The most common dietary phytosterols are β-sitosterol, campesterol and stigmasterol. Among these, the most abundant phytosterol is β-sitosterol. Studies have shown that β-sitosterol exhibits anti-inflammatory, angiogenic and immune-modulating properties [10]. β-sitosterol is reported to activate Fas signaling in breast cancer cells [11], and induce cell cycle arrest and apoptotic cell death in prostate cancer cells [12,13].

Herein, we evaluated the efficacy of β-sitosterol on three different cancer cell lines including human skin epidermoid carcinoma cell line (A431), human lung epithelial carcinoma cell line (A549) and human breast adenocarcinoma cell line (MDA-MB-231). It was observed that as compared to A431 and A549 cells, β-sitosterol showed prominent growth inhibitory and pro-apoptotic activity in MDA-MB-231 cells. Further, study provides valuable insight into the chemopreventive efficacy and associated molecular alterations of β-sitosterol in breast cancer cells in culture.

## Methods

### Cell lines and reagents

**Figure 1:**
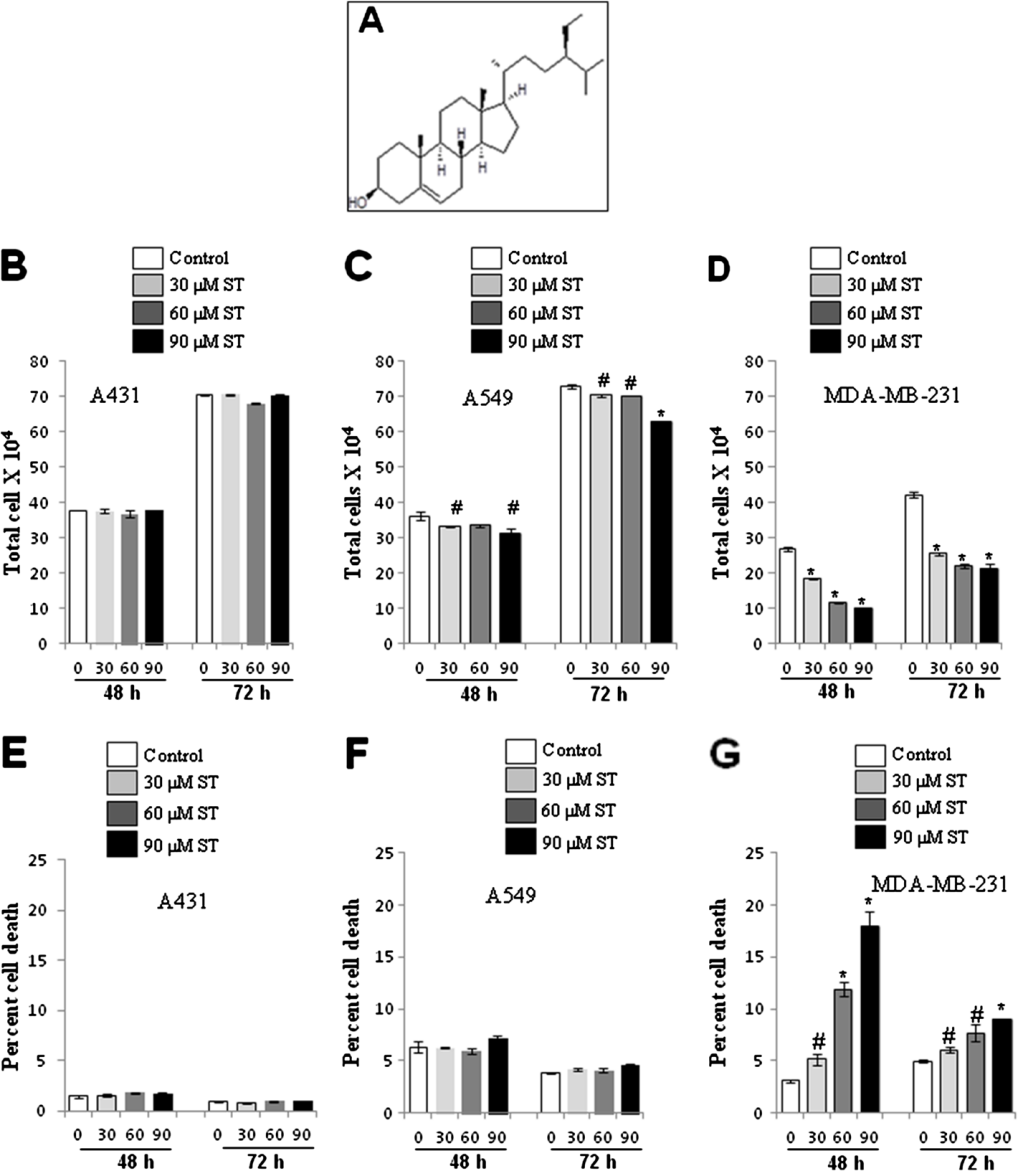
**Effect of β-sitosterol (ST) on growth and cell death in human cancer cells. (A)** Chemical structure of β-sitosterol, Chemical formula:17-(5-Ethyl-6- methylheptan-2-yl)-10,13-dimethyl-2,3,4,7,8,9,11,12,14,15,16,17-dodecahydro-1H cyclopenta[a]phenanthren-3-ol. Cells were plated overnight and treated with either DMSO control or 30, 60 and 90 μM β-sitosterol for 48 and 72 h. At the end of treatment, cells were collected and counted with hemocytometer after staining with trypan blue dye using phase contrast inverted microscope for total cell number of **(B)** A431, **(C)** A549 and **(D)** MDA-MB-231; and percent dead cells of **(E)** A431, **(F)** A549 and **(G)** MDA-MB-231. Data shown are mean ± S.E. of triplicate samples for each treatment. #, p < 0.05; $, p < 0.01; *, p < 0.001 compared with the respective controls.

A431 and A549 cells were from ATCC (Manassas, USA) and MDA-MB-231 cells were from NCCS Pune, India. These cells were cultured in DMEM, RPMI-1640 and L-15, respectively, each with 10% fetal bovine serum, 100 units/ml penicillin and 100 μg/ml streptomycin at 37°C in a humidified 95% air and 5% CO_2_ atmosphere. β-sitosterol (ST) was obtained from Sigma Chemical Co. (St. Louis, MO, USA; Figure 1A). ST was dissolved in DMSO and an equal amount (0.1% v/v) of DMSO was present in all the treatment groups including control. Anti-CDK4, cyclin D1 antibodies were from Santa Cruz Biotechnology (CA, USA); anti-beta-actin antibody was from Sigma; and anti-p21/Cip1, p27/Kip1, phospho and total Erk1/2 and Akt, and HRP-conjugated secondary antibodies were from Cell Signaling Technology (MA, USA).

### Cell growth and death assays

A431, A549 and MDA-MB-231 cells (1 × 10^5^) were plated in 60 mm culture dishes, and next day fed with fresh medium and treated with different doses of ST (30, 60 and 90 μM) in complete medium. After 48 and 72 h of these treatments, total cells were collected by brief trypsinization, and washed with PBS. Total cell number was determined by counting each sample in duplicate using a hemocytometer under an inverted phase contrast microscope (Carl Zeiss) using trypan blue dye. Dead cells could not exclude the dye and showed blue staining of the cell. Each treatment for each time point was done in triplicates. Experiments were repeated at least two-three times.

### Cell cycle analysis

A431, A549 and MDA-MB-231 cells were grown at similar confluency as in cell growth assay, and treated with desired doses of ST (0, 30, 60 and 90 μM) in complete medium for 48 and 72 h. At the end of each treatment time, cells were collected after a brief incubation with trypsin–EDTA followed by processing for cell cycle analysis as reported earlier [14]. Briefly, 0.5x10^5^ cells were suspended in 0.5 ml of saponin/propidium iodide (PI) solution [0.3% saponin (w/v), 25 μg/ml PI (w/v), 0.1 mM EDTA and 10 μg/ml RNase (w/v) in PBS], and incubated over night at 4°C in dark. Cell cycle distribution was then analyzed by flow cytometry using FACS Aria III flow cytometer (BD Biosciences, San Jose, CA, USA) at Central Instrument Facility, Central University of Gujarat, Gandhinagar.

### Annexin V apoptosis assay

To quantify ST-induced apoptotic death of MDA-MB-231 cells, annexin V/PI staining was performed followed by flow cytometry, as described earlier [15]. Cells were seeded and treated with 0, 60 and 90 μM of ST for 48 h. Briefly, after treatment, both floating and attached cells were pooled and subjected to annexin V/PI staining using FITC Annexin V Apoptosis Detection Kit 1 from BD Biosciences, USA and following the step-by-step protocol provided by the manufacturer.

### Analysis of mitochondrial transmembrane potential (ΔΨ m)

To analyse mitochondrial transmembrane potential and determine JC-1 monomer/ JC-1 dimer ratio, MDA-MB-231 cells were seeded and treated with 0, 60 and 90 μM of ST for 24 h. Cells were harvested, volume was adjusted to 1 ml for 2 × 10^5^ cells by adding pre-warmed (37°C) fresh cell culture medium, 2.5 μl JC-1 dye was added from 5 mg/ml JC-1 dye stock to cell suspension. Then cell suspension was gently mixed until dye got properly dissolved and incubated for 10 min in dark at 37°C. Cell suspension was washed with 2 ml 1X PBS and centrifuged at 1500 rpm for 5 min, discarded the supernatant and repeated the wash. Cells were resuspended in 0.3 ml 1X PBS. Subsequently, cells were analyzed with BD FACS Aria III flow cytometer as reported earlier [16]. Data was analyzed as JC-1 monomer/dimer ratio.

### Immunoblot analysis

MDA-MB-231 cells were treated with ST (0, 60, and 90 μM) for 48 h and whole cell lysates were prepared as described [17]. Protein estimation was done by Bradford method. Lysates (60–80 μg/sample) were denatured in SDS-PAGE sample buffer and boiled on water bath for 5 min. Samples were loaded onto 12% denaturing SDS-PAGE gels and proteins resolved at constant voltage. Proteins from the gel were transferred onto nitrocellulose membrane using transfer assembly. Membranes were blocked in blocking buffer for 1 h and incubated with specific primary antibodies followed by appropriate HRP-linked secondary antibody and processed for ECL detection. Bands on films were scanned using high resolution scanner [18].

### Statistical analysis

All statistical analyses were carried out with Sigma Stat software version 2.03 (Jandel Scientific, San Jose, CA, USA). Student’s *t*-test was used for comparing the control group with treatment groups for statistical significance. *P* < 0.05 was considered significant. All the experiments were repeated at least twice with similar results.

## Results

### Effect of β-sitosterol on growth and death of cancer cells

Trypan blue is a diazo dye which can not be excluded from the dead cells. This method is also known as a dye exclusion method. This test is used to determine the number of viable cells present in a cell suspension. It is based on the principle that live cells possess intact cell membranes that exclude dyes such as trypan blue, whereas in dead cells the dye is not excluded out. In this test, a cell suspension is mixed with dye and then microscopically examined for live and dead cells. The viable cells will have a clear cytoplasm whereas non-viable cells will have blue colour. A431 cells were treated in exponential growth phase with different doses of ST (30, 60 and 90 μM) for 48 and 72 h. There was no significant difference between the control and ST treatments in total cell number (Figure 1B). We also did not observe any considerable difference in cell death of A431 cells after ST treatments (Figure 1E). In case of A549 cells, there was a significant decrease in total cell number by 8.2% (p ≤ 0.05) to 13.3% (p ≤ 0.05) in 48 h and 3.4% (p ≤ 0.05) to 13.7% (p ≤ 0.0001) in 72 h (Figure 1C). The increase in cell death of A549 cells was not significant except at 72 h of 90 μM ST treatment (Figure 1F).

In case of MDA-MB-231 cells, there was a strong and significant cell growth inhibition which accounted for 31% (p ≤ 0.0001) to 63% (p ≤ 0.0001) in 48 h and 40% (p ≤ 0.0001) to 50% (p ≤ 0.0001) in 72 h (Figure 1D). The increase in cell death was about 2% (p ≤ 0.05) to 15% (p ≤ 0.0001) in 48 h and 1% (p ≤ 0.05) to 4% (p ≤ 0.0001) in 72 h of ST treatments as compared to their respective controls (Figure 1G). These results suggested better antiproliferative and cell death inducing effect of ST on MDA-MB-231 breast cancer cells as compared to skin carcinoma A431 and lung carcinoma A549 cells.

### Effect of β-Sitosterol on cell cycle progression of cancer cells

Cell cycle analysis was done by quantitation of DNA content using propidium iodide fluorescent dye for each phases of the cell cycle by flow cytometry. The fluorescence intensity of the stained cells at certain wavelengths will therefore correlate with the amount of DNA they contain. A431 cells were treated with different doses of ST (30, 60 and 90 μM) for 48 and 72 h. Cell cycle distribution analysis of A431 cells showed that, there were 3% and 5% increases in G2/M phase of cell population (Additional file 1: Figure S1A) in 48 and 72 h of ST treatments, respectively. A549 cells showed 4% increase in S phase in 48 h of ST treatments (Additional file 1: Figure S1B). MDA-MB-231 cells showed 5% and 3% increases in G1 phase in 48 h and 72 h of ST treatments, respectively (Additional file 1: Figure S1C). Overall, only a moderate effect of ST was observed on cell cycle progression of cancer cells.

### Effect of β-Sitosterol on G0/G1 phase cell cycle protein levels in MDA-MB-231 cells

**Figure 2:**
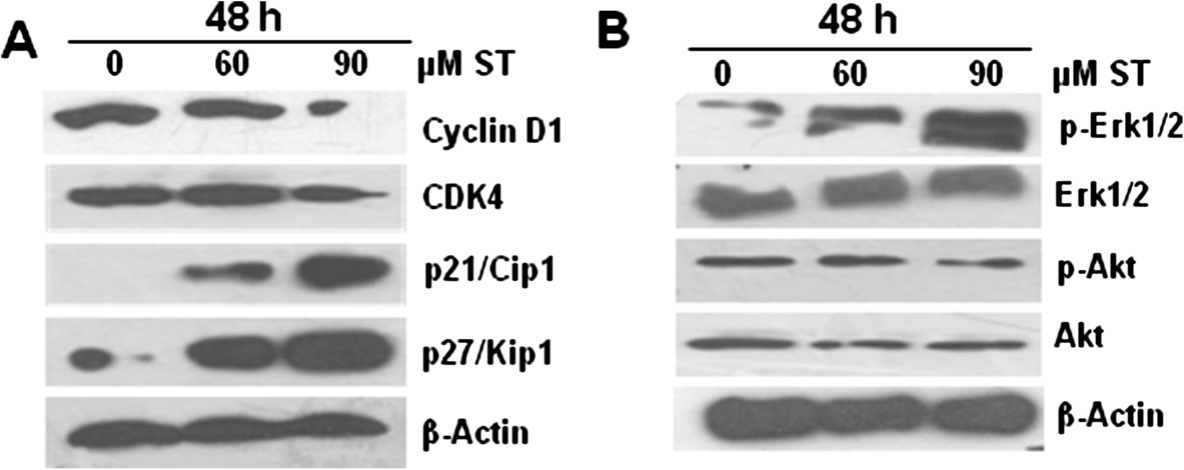
**Effect of β-sitosterol (ST) on G0/G1 phase cell cycle regulators and mitogenic and survival signaling in breast cancer cells.** MDA-MB-231 cells were treated with either DMSO control or various doses of β-Sitosterol (60 and 90 μM) for 48 h. At the end of these treatments, cell lysate was prepared and western blot analysis was performed. Membranes were probed with **(A)** anti-cyclin D1, CDK-4, p21/Cip1, p27/Kip1, and **(B)** anti-p-Erk1/2, Erk1/2, p-Akt and Akt antibodies followed by peroxidase-conjugated appropriate secondary antibodies, and visualized by ECL detection system. Membranes were striped and re-probed with anti-β actin for loading control.

Western blot analysis was done to study the molecular effect of ST on cell cycle regulatory molecules including cyclin-dependent kinase (CDK), Cyclin and CDKI (CDK inhibitor), which were involved in the G0/G1 arrest. Since we observed a moderate G0/G1 arrest in MDA-MB-231 cells, we analysed these cells after ST (60–90 μM) of 48 h that showed a dose-dependent decrease in CDK4 and cyclin D1 protein levels with stronger effect at higher concentration (Figure 2A). We also examined the effect of ST treatment on negative regulator of cell cycle, i.e. (CDKI) such as p21/Cip1 and p27/Kip1, that controls the activity of CDK through various phases of cell cycle, including G0/G1. We observed an increase in p21/Cip1 and p27/Kip1 protein levels which were relatively higher at 90 μM concentration of ST and may explain the effect for G0/G1 arrest compared to control (Figure 2A). These results suggest that ST induced G0/G1 arrest in MDA-MB-231 cells could be mediated *via* modulation of CDK-cyclin-CDKI protein levels.

### Effect of β-Sitosterol on Erk1/2 and Akt activation in MDA-MB-231 cells

After 48 h of ST treatment we observed a dose-dependent increase in Erk1/2 phosphorylation without any change in its total protein level (Figure 2B). However, we did not observe any considerable change in protein levels of p-Akt and total Akt as compared to control (Figure 2B). These results suggest that ST may preferentially activate Erk1/2 signaling for its growth inhibitory and cell death inducing effects on MDA-MB-231 cells.

### Effect of β-Sitosterol on apoptotic cell death in MDA-MB-231 cells

**Figure 3:**
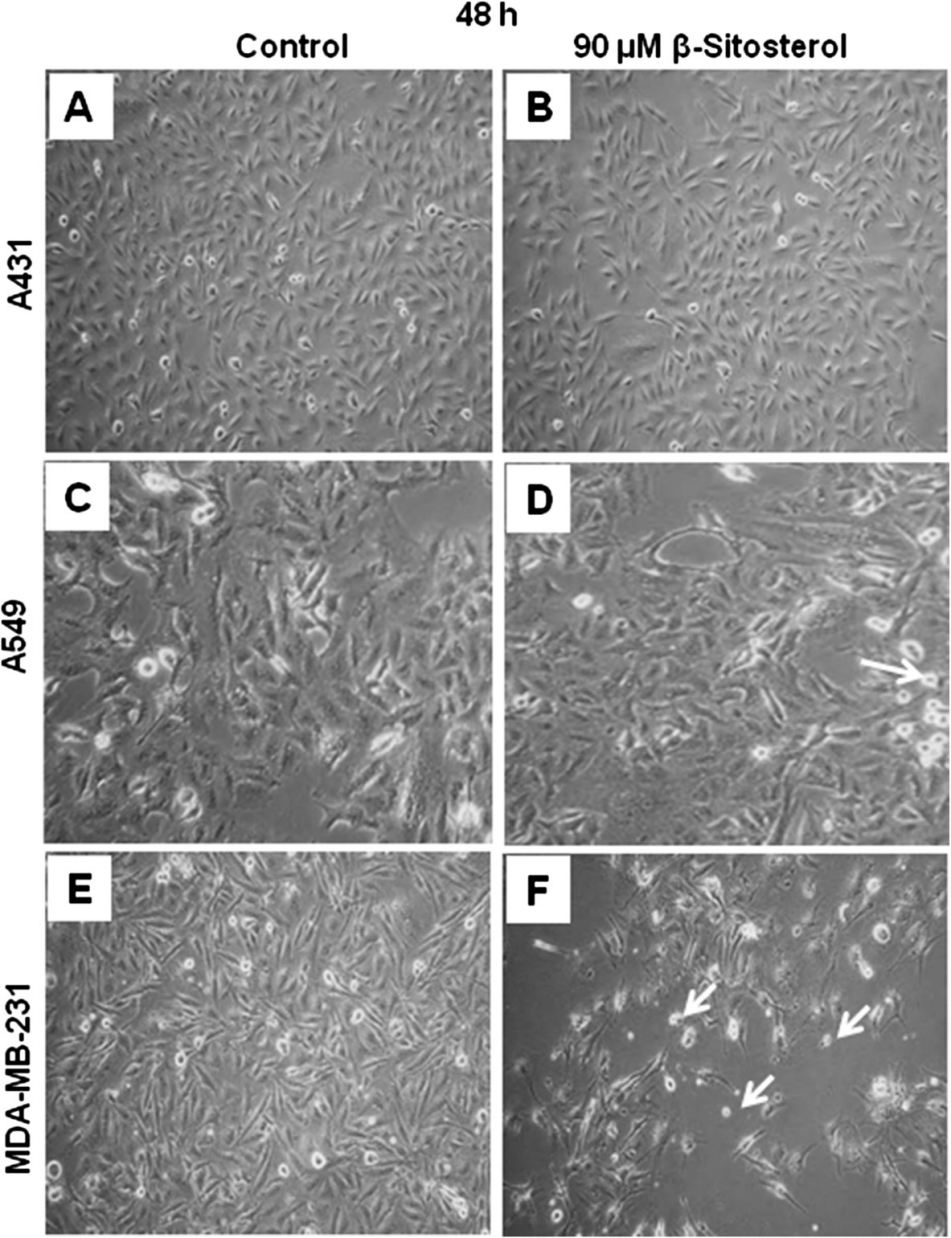
**Effect of β-sitosterol (ST) on cell morphology of human skin epidermoid carcinoma, human lung epithelial carcinoma and human breast carcinoma cells. (A & B)** A431, **(C & D)** A549, and **(E & F)** MDA-MB-231 cells were treated with 90 μM ST for 48 h. A, C and E represents untreated cells whereas B, D and F represents the cells treated with ST. Arrows indicate the shrinkage of cell with condensed nuclei which may apoptotic in nature.

Apoptosis is a cell death process characterized by morphological and biochemical features occurring at different stages. The cells undergoing apoptosis translocate phosphatidyl serine to the outer layer of the membrane. This occurs in the early phases of apoptotic cell death during which the cell membrane remains intact [19]. The morphology of MDA-MB-231 cells as compared to A431 and A549 cells after 48 h of ST treatment suggests that cells may undergo apoptosis (Figure 3). To investigate this possibility MDA-MB-231 cells were treated with 60 and 90 μM of ST for 48 and 72 h, and stained with FITC-annexin V and analyzed by flow cytometry. There was up to 2-fold (p ≤ 0.05) increase in apoptotic cell population following ST treatment (data not shown).

**Figure 4:**
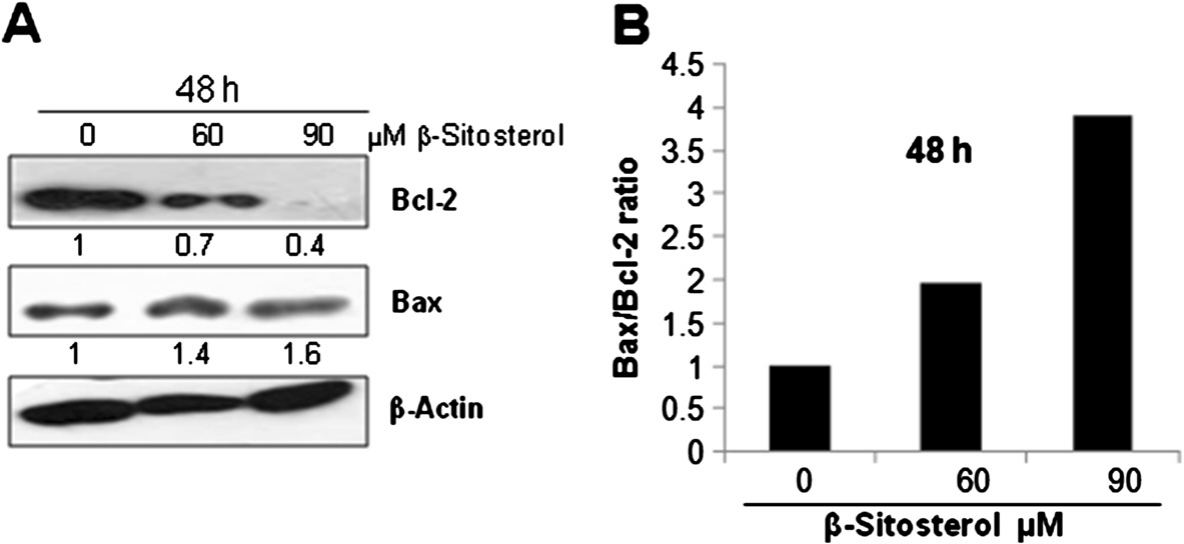
**Effect of β-sitosterol (ST) on apoptosis and Bax/Bcl-2 ratio in breast carcinoma cells.** MDA-MB-231 cells were treated with DMSO (control) or 60 and 90 μM ST for 48 h. **(A)** Western blot analyses were performed to examine the expression level of Bax and Bcl-2 proteins. Membranes were probed with anti-Bax and Bcl-2 antibodies followed by peroxidase-conjugated appropriate secondary antibodies, and visualized by ECL detection system. Membrane was striped and re-probed with anti-β actin for loading control. Densitometric value of bands are shown below each band as fold change from that of control and corrected with loading control **(B)** Represents the ratio of Bax/Bcl-2 protein expression levels.

Bcl-2 is anti-apoptotic protein whereas Bax is a pro-apoptotic protein, and ration of both is known to determine the apoptotic response of the cell. To investigate the anti-apoptotic and pro-apoptotic gene expression, western blotting was performed against Bcl-2 and Bax proteins. A concentration-dependent decrease was observed in Bcl-2 protein level whereas Bax showed a moderate concentration-dependent increase after 48 h of ST treatment (Figure 4A). There was a 2–4 fold increase in Bax/Bcl-2 ratio (Figure 4B), suggesting that induction of apoptosis involving mitochondria could be one of the mechanisms of cell death induced by ST.

### Effect of β-Sitosterol mitochondrial membrane potential in MDA-MB-231 cells

The loss of mitochondrial membrane potential is a hallmark for apoptosis [20]. JC-1(5,5″,6,6″- tetrachloro-1,1″,3,3″-tetraethylbenzimidazolylcarbocyanineiodide) is used mostly for detecting mitochondrial depolarization occurring in the early stages of apoptosis. It exhibits potential-dependent accumulation in mitochondria, indicated by a fluorescence emission shift from green to red. In non-apoptotic cells, JC-1 exists as dimer and accumulates as aggregates in the mitochondria which stains red. Whereas, in apoptotic and necrotic cells, JC-1 exists in monomeric form in cytosol and stains the cytosol green. Changes in the mitochondrial membrane potential (ΔΨ) were analyzed using JC-1 dye.

**Figure 5:**
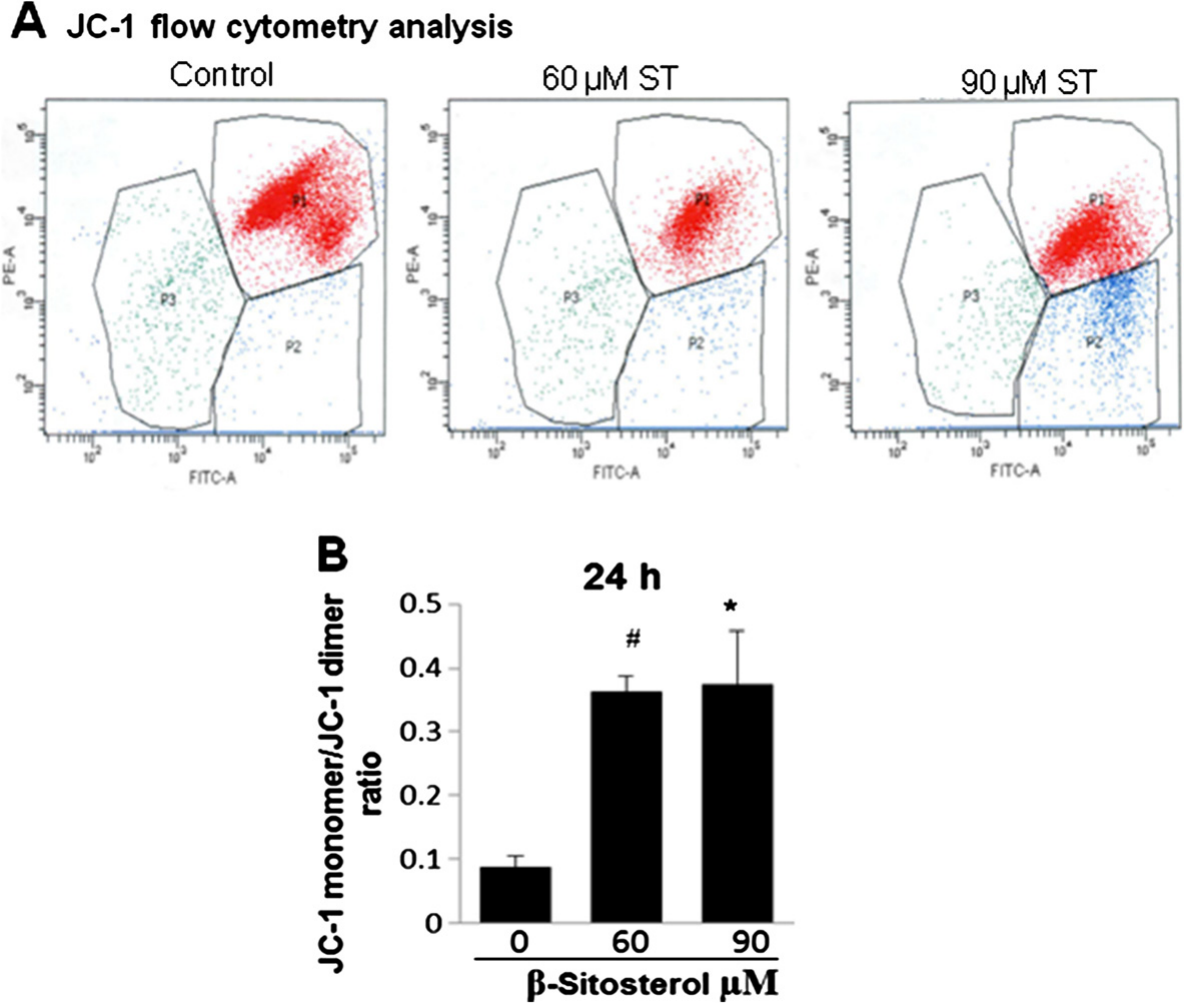
**Effect of β-sitosterol (ST) on mitochondrial membrane potential in breast carcinoma cells.** MDA-MB-231 cells were treated with DMSO (control) or 60 and 90 μM ST for 24 h. **(A)** Mitochondrial membrane potential was measured by JC-1 staining and analyzed by flow cytometry as described in Methods. **(B)** The quantitative data is presented as means of 590 nm/520 nm (P2/P1) emission spectrum. The data shown are mean ± S.E. of triplicate samples for each treatment. #, p < 0.05; *, p < 0.001 compared with control.

Cells were similarly seeded and treated as in apoptosis assay. After treatment cells were harvested and processed for JC-1 staining and subjected to flow cytometry. Red to green shift suggests the shift from JC-1 dimer to JC-1 monomer (Figure 5A). Results show that there was a significant increase in monomer/dimer ratio which accounted for 4.5 fold increase (p ≤ 0.05) at 60 μM ST, and 5-fold increase (p ≤ 0.001) at 90 μM ST after 24 h of treatment (Figure 5B). This result suggests that ST causes depolarization of mitochondrial membrane potential which could be linked with the induction of apoptosis in breast cancer cells.

## Discussion

Chemoprevention is a promising strategy which involves intervention by natural and synthetic agents at early stages in the disease process or in high risk individuals to reduce the development of cancer [21]. This strategy is promising for reducing cancer incidence both in well-defined high-risk groups of people as well as in the general population. There is an urgent need to explore potent, non-toxic and less expensive chemopreventive agents that inhibit cancer growth and progression.

Our present investigation of ST against human skin epidermoid carcinoma cell line A431, human lung epithelial carcinoma cell line A549 and human breast adenocarcinoma cell line MDA-MB-231 is an effort to find its anticancer efficacy in different epithelial cancers. The results of cell counting data clearly show that ST does not have any significant effect on cell growth, cell death and cell cycle progression in A431 cells. However, a minor but considerable effect was observed only for cell growth inhibition without any effect on cell death or cell cycle progression in A549 cells. In case of MDA-MB-231 cells, ST showed a significant growth inhibition, cell death and cell cycle arrest. These observations suggest that ST has differential efficacy for skin, lung and breast cancer cells which is in order of A431 < A549 < <MDA-MB-231.

The cancer chemopreventive strategies include the targeting of limitless replication potential and deregulated cell cycle progression in cancer cells [22]. Cell cycle progression is mediated by the activity of cyclin-dependent kinases (CDK) in complex with regulatory subunit cyclins [23]. A frequent target in carcinogenesis is the deregulation of G1 to S phase progression of the cell cycle [24]. Cyclin D1 is a key regulatory protein at G1/S checkpoint of the cell cycle that forms complexes with CDK4 or CDK6. It is considered as an oncogene, and overexpressed in many cancers, including prostate, breast, esophagus, lung, head and neck and colon [25,26]. It acts as a growth sensor and provides a link between mitogenic stimuli and the cell cycle. CDK4 is a key factor in promoting the initiation and development of tumors [27], and amplified and over expressed in a number of human tumors including the gliomas, sarcomas, breast tumors and colorectal carcinomas [28]. Since we observed considerable G1 phase arrest in MDA-MB-231 cells, we also did analysis for the cyclin D1 and CDK4 protein levels which were decreased after ST treatment. This suggests that down-regulation of cyclin D1 and CDK4 may be associated with ST-induced G1 phase arrest in breast carcinoma cells.

Cyclin-dependent kinase inhibitors (CDKI) are tumor suppressor proteins that bind to active CDK-cyclin complexes and inhibit their kinase activities [29]. p21/Cip1 is a universal CDKI and plays a critical role in the cellular response for cell cycle arrest [30]. p27/Kip1 is another important member of CDKI which connects the anti- proliferative signals for cell cycle arrest [31]. Lower level of p27/Kip1 protein is known as a prognostic marker for many cancers [32].

Our study showed that ST increases p21/Cip1 and p27/Kip1 protein levels in MDA-MB-231 cells, and thus it can also take part in inhibiting the kinase activity of CDK. Together, ST-induced G1 arrest could be mediated *via* down-regulation of CDK 4 and cyclin D1, and up regulation of p21/Cip1 and p27/Kip1 in MDA-MB-231 cells. Furthermore, the changes observed at molecular level suggest that these CDKI might be having alternative mechanism/s such as apoptosis, other than their cell cycle effect, however, further studies are needed to explore such assumptions.

Apoptosis is suggested as one of the major mechanisms for the targeted therapy of various cancers including breast cancer [33]. Most of the presently available cytotoxic anticancer drugs mediate their effect *via* apoptosis in cancer cells [34]. Changes in negatively charged phosphatidyl serine (PS) asymmetry in cell membrane is analyzed by its binding with annexin V conjugated with FITC by flow cytometry. Annexin V analysis suggests that ST caused cell death is mainly because of apoptosis induction in MDA-MB-231 cells. The longer exposure of lower dose (60 uM) of ST showed time-dependent effect for apoptosis; however, the higher dose (90 uM) achieved its maximum effect at 48 h after the treatment. Next, we explored the molecular alterations associated with apoptosis.

The Bcl-2 family includes both anti–apoptotic (e.g., Bcl-2 itself) and pro–apoptotic (e.g., Bax) proteins. Their expression levels, interaction, ratio, and translocation of different members determine the overall pro–apoptotic or anti–apoptotic fate of the cell [35]. The increased expression of Bax is often associated with the increased apoptosis whereas the increased expression of Bcl-2 is associated with the inhibition of apoptosis and cell survival mechanisms [36]. Western blotting analysis showed a decrease in expression level of Bcl-2 and an increase in expression level of Bax. Furthermore, there was a strong increase in Bax to Bcl-2 ratio, which is known to favor apoptosis [36] suggesting that ST caused increase in Bax/Bcl-2 ratio may play a significant role in induction of apoptosis in MDA-MB-231 cells. To further investigate the molecular changes associated with apoptosis induction by ST in MDA-MB-231 cells, mitochondrial membrane potential was measured. ST caused the depolarization of mitochondrial membrane potential which is associated with the induction of an early apoptotic event leading to mitochondrial apoptosis in cells.

## Conclusions

The current study shows that β-Sitosterol (ST), a dietary phytosterol has stronger anticancer activity against breast cancer cells compared to lung and skin cancer cells which may be attributed to the differential expression of genes including hormones, receptors and tissue specific proteins (Additional file 2: Figure S2). Along with anti-proliferative and growth inhibitory effect, ST induced G0/G1 cell cycle arrest *via* modulation of cell cycle regulators CDK4, cyclin D1, p21/Cip1and p27/Kip1 breast cancer cells. Further ST caused cell death involves the induction of apoptosis in breast cancer cells *via* mitochondrial membrane depolarization and increase in Bax/Bcl-2 ratio. This study provides valuable insight into the chemopreventive efficacy and associated molecular alterations of ST in breast cancer cells. However, further studies are needed to understand and assess the potential clinical utility of ST as a chemopreventive agent against breast cancer.

## Abbreviations

ST: β-sitosterol; CDK: Cyclin-dependent kinase; CDKI: Cyclin-dependent kinase inhibitor; FACS: Fluorescence-activated cell sorting.

## Competing interests

The authors declare that they have no competing interests.

## Authors’ contributions

SSV has done the experiment and written the manuscript; RKK has contributed in developing of hypothesis and designing of experiment; RPS has developed hypothesis, designed experiments, written the manuscript and overall supervised the study. All authors read and approved the final manuscript.

## Pre-publication history

The pre-publication history for this paper can be accessed here:

http://www.biomedcentral.com/1472-6882/13/280/prepub

## Supplementary Material

Additional file 1: Figure S1 Effect of β-sitosterol (ST) on cell cycle progression in cancer cells. **(A)** A431, **(B)** A549 and **(C)** MDA-MB-231 cells were treated with either DMSO control or various doses of β-Sitosterol (60 and 90 μM) for 48 and 72 h. Cell cycle analysis was performed at the end of each treatment as detailed in Methods. Click here for file

Additional file 2: Figure S1 Differential effects of β-sitosterol on human cancer cell lines. β-sitosterol up-regulates CDKIs, p21 and p27 and down-regulates cyclin D1 and CDK4 to induce G1 arrest in breast adenocarcinoma MDA- MB-231 cells. On the other hand, it causes an increase in Bax/Bcl-2 ratio and mitochondrial membrane depolarization to induce apoptosis in breast cancer cells. Click here for file
