# Supplementary material for: β-sitosterol induces G1 arrest and causes depolarization of mitochondrial membrane potential in breast carcinoma MDA-MB-231 cells
Source: BMC Complement Altern Med. 2013 Oct 25;13:280. doi: 10.1186/1472-6882-13-280 (PMC3819702; doi:10.1186/1472-6882-13-280)
Supplement: Additional file 1: Figure S1 — Effect of β-sitosterol (ST) on cell cycle progression in cancer cells. (A) A431, (B) A549 and (C) MDA-MB-231 cells were treated with either DMSO control or various doses of β-Sitosterol (60 and 90 μM) for 48 and 72 h. Cell cycle analysis was performed at the end of each treatment as detailed in Methods. [file 1472-6882-13-280-S1.ppt]

## Slide 1
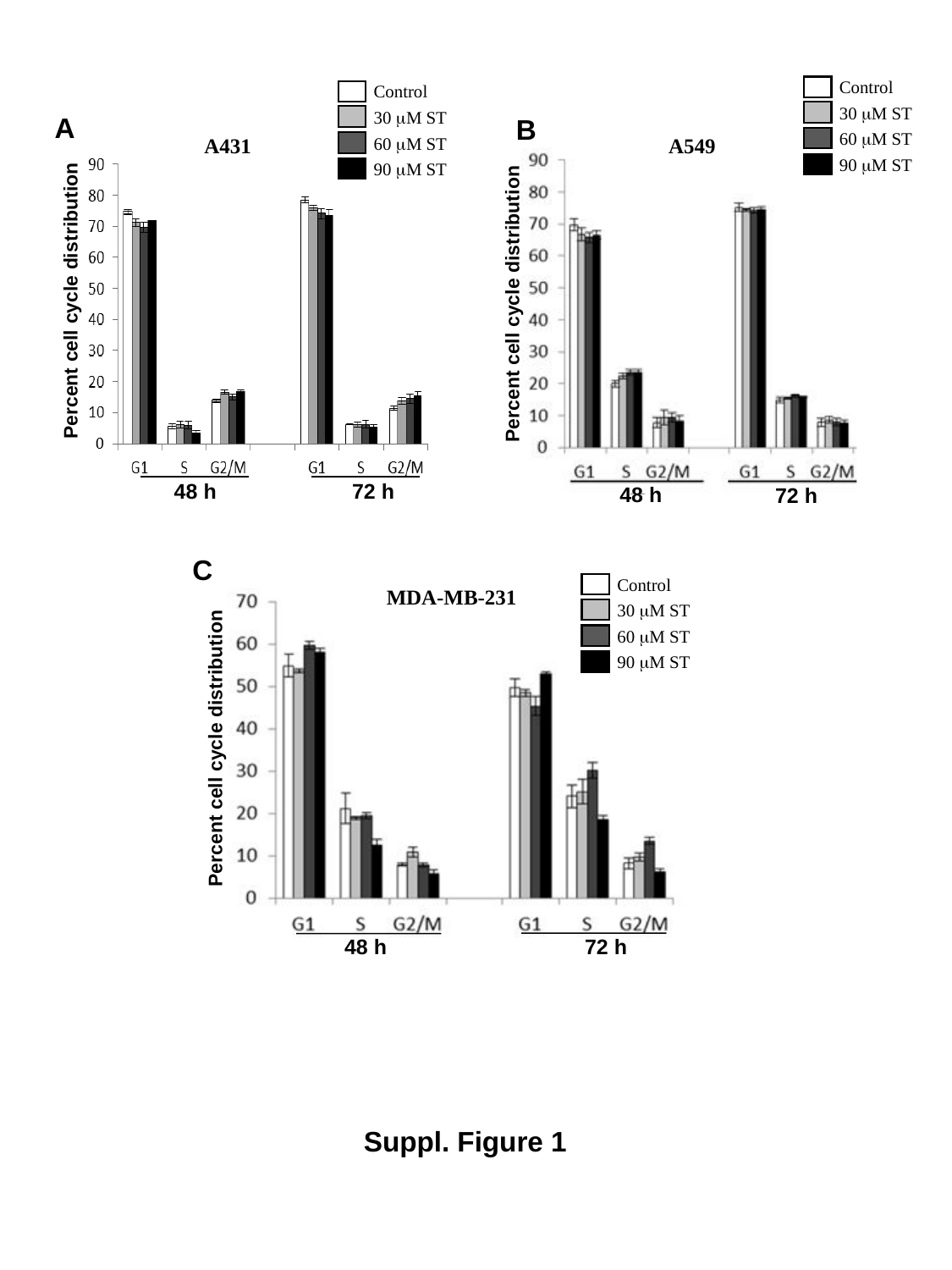

Control
30 μM ST
60 μM ST
90 μM ST
Control
30 μM ST
60 μM ST
90 μM ST
A
B
A549
A431
Percent cell cycle distribution
Percent cell cycle distribution
48 h
72 h
48 h
72 h
C
Control
30 μM ST
60 μM ST
90 μM ST
MDA-MB-231
Percent cell cycle distribution
48 h
72 h
Suppl. Figure 1
