# Supplementary material for: β-sitosterol induces G1 arrest and causes depolarization of mitochondrial membrane potential in breast carcinoma MDA-MB-231 cells
Source: BMC Complement Altern Med. 2013 Oct 25;13:280. doi: 10.1186/1472-6882-13-280 (PMC3819702; doi:10.1186/1472-6882-13-280)
Supplement: Additional file 2: Figure S1 — Differential effects of β-sitosterol on human cancer cell lines. β-sitosterol up-regulates CDKIs, p21 and p27 and down-regulates cyclin D1 and CDK4 to induce G1 arrest in breast adenocarcinoma MDA- MB-231 cells. On the other hand, it causes an increase in Bax/Bcl-2 ratio and mitochondrial membrane depolarization to induce apoptosis in breast cancer cells. [file 1472-6882-13-280-S2.ppt]

## Slide 1
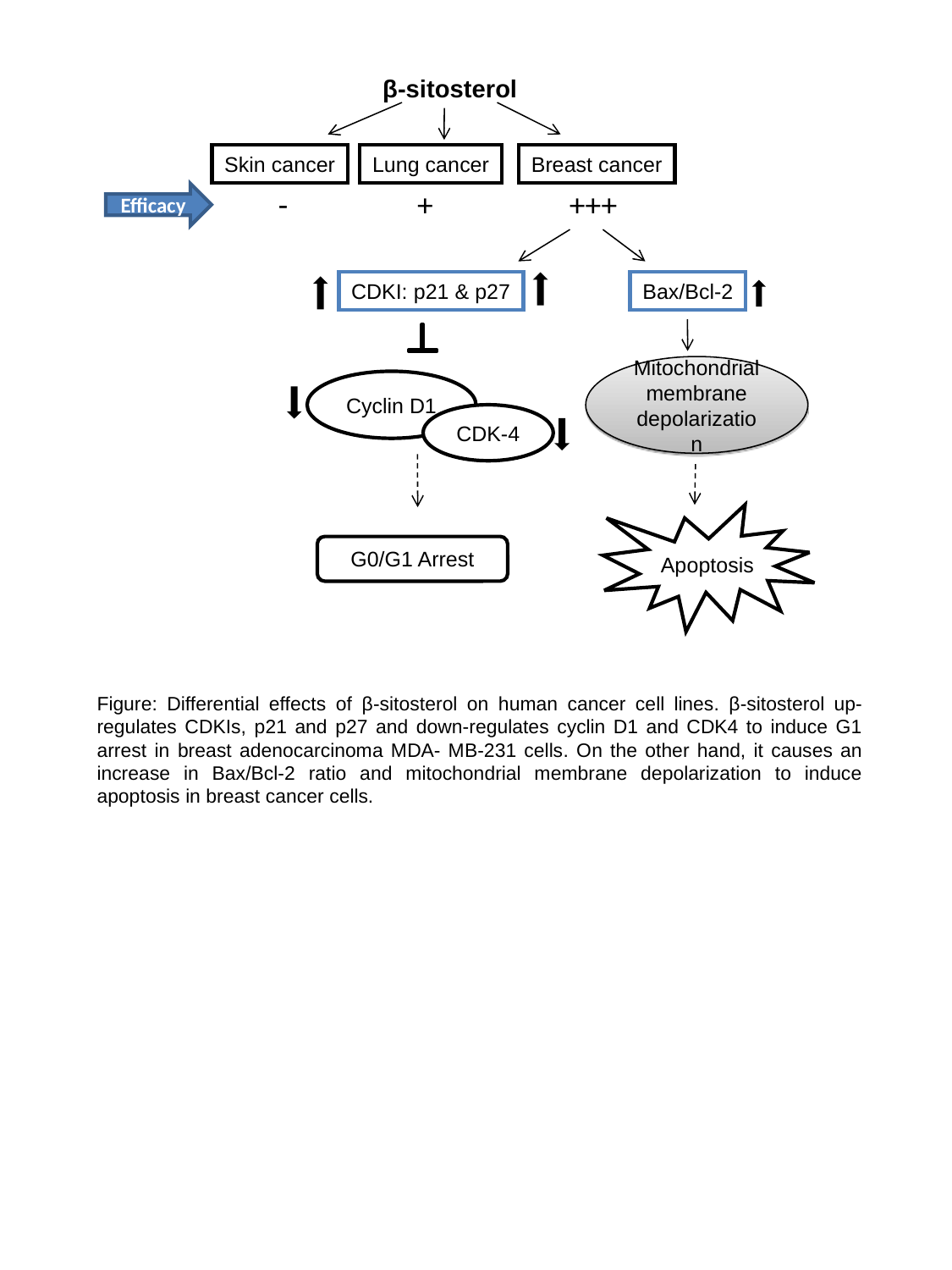

β-sitosterol
Skin cancer
Lung cancer
Breast cancer
 - + +++
Efficacy
CDKI: p21 & p27
Bax/Bcl-2
Mitochondrial membrane depolarization
Cyclin D1
CDK-4
Apoptosis
G0/G1 Arrest
Figure: Differential effects of β-sitosterol on human cancer cell lines. β-sitosterol up-regulates CDKIs, p21 and p27 and down-regulates cyclin D1 and CDK4 to induce G1 arrest in breast adenocarcinoma MDA- MB-231 cells. On the other hand, it causes an increase in Bax/Bcl-2 ratio and mitochondrial membrane depolarization to induce apoptosis in breast cancer cells.
